# CENPF/CDK1 signaling pathway enhances the progression of adrenocortical carcinoma by regulating the G2/M-phase cell cycle

**DOI:** 10.1186/s12967-022-03277-y

**Published:** 2022-02-05

**Authors:** Yu-gang Huang, Dan Li, Li Wang, Xiao-min Su, Xian-bin Tang

**Affiliations:** 1 Department of Pathology, Taihe Hospital, Hubei University of Medicine, Shiyan, 442000 China; 2 Department of Immunology, Nankai University School of Medicine, Tianjin, 300110 China

**Keywords:** Adrenocortical carcinoma, ACC, CENPF, CDK1, p53, Cell cycle

## Abstract

**Background:**

Adrenocortical carcinoma (ACC) is an aggressive and rare malignant tumor and is prone to local invasion and metastasis. And, overexpressed Centromere Protein F (CENPF) is closely related to the oncogenesis of various neoplasms, including ACC. However, the prognosis and exact biological function of CENPF in ACC remains largely unclear.

**Methods:**

In the present essay, the expression patterns and prognostic value of CENPF in ACC were investigated in clinical specimens and public cancer databases, including GEO and TCGA. The potential signaling mechanism of CENPF in ACC was studied based on gene-set enrichment analysis (GSEA). Furthermore, a small RNA interference experiment was conducted to probe the underlying biological function of CENPF in the human ACC cell line, SW13 cells. Lastly, two available therapeutic strategies, including immunotherapy and chemotherapy, have been further explored.

**Results:**

The expression of CENPF in human ACC samples, GEO, and TCGA databases depicted that CENPF was overtly hyper-expressed in ACC patients and positively correlated with tumor stage. The aberrant expression of CENPF was significantly correlated with unfavorable overall survival (OS) in ACC patients. Then, the GSEA analysis declared that CENPF was mainly involved in the G2/M-phase mediated cell cycle and p53 signaling pathway. Further, the in vitro experiment demonstrated that the interaction between CENPF and CDK1 augmented the G2/M-phase transition of mitosis, cell proliferation and might induce p53 mediated anti-tumor effect in human ACC cell line, SW13 cells. Lastly, immune infiltration analysis highlighted that ACC patients with high CENPF expression harbored significantly different immune cell populations, and high TMB/MSI score. The gene-drug interaction network stated that CENPF inhibitors, such as Cisplatin, Sunitinib, and Etoposide, might serve as potential drugs for the therapy of ACC.

**Conclusion:**

The result points out that CENPF is significantly overexpressed in ACC patients. The overexpressed CENPF predicts a poor prognosis of ACC and might augment the progress of ACC. Thus, CENPF and related genes might serve as a novel prognostic biomarker or latent therapeutic target for ACC patients.

**Supplementary Information:**

The online version contains supplementary material available at 10.1186/s12967-022-03277-y.

## Background

Adrenocortical carcinoma (ACC), as an invasive and rare malignant neoplasm that originated from the adrenal cortex, is prone to local invasion and metastasis through blood and lymph nodes, accounting for 14% of primary adrenal incidentalomas [1]. The overall incidence of ACC reaches a peak in childhood and adults (40–60 years old) with a bimodal age distribution. In the United States, general cancer incidence is about 4500 cases/million/year in adults and 150 cases/million/year in children [2, 3]. The median overall survival is about 3.5 years, and the 5-year survival rate is about 35% for ACC with locally advanced stages, and 0–28% for ACC with metastases [4, 5]. It indicates that ACC is characterized by tumor heterogeneity and harbors a dismal prognosis. At present, effective drugs for the therapies of ACC are rare. From 1914 to now, surgery and mitotane/platinum-based chemotherapy are still the only available or effective therapeutic strategies [1]. ACC is still a homogeneous class with unique biological characteristics from a pan-cancer landscape, even if ACC emerges heterogeneous molecular profiles. Hitherto, several biomarkers associated with diagnosis, prognosis, or therapy, which might exert vital roles in determining the aggressiveness of ACC, have been reported. Molecular studies have demonstrated that TP53 [6, 7] and CTNNB1 [8] mutations serve as the most common driver genes for ACC patients. About 10% ACC patients from the TCGA datasets had a pathogenetic TP53 variant [8]. Sbiera et al. proposed that SOAT1 may serve as molecular target of mitotane in ACC [9]. Accumulative literatures have demonstrated that inhibition of WNT–β-catenin signaling pathway [10], cell-cycle progression through down-regulation of p53–RB [11], defects in mismatch repair related enzymes (dMMR) [12], DNA methylation [13], and abnormal maintenance of telomere (ATRX [14, 15], DAX [16] and TERT [8, 17]), were closely bound up with the deterioration and poor prognosis of ACC. In addition, the unique feature of ACC is steroid differentiation, which has been viewed as a potential therapeutic target. Some drugs are undergoing preclinical or clinical research [18]. Despite the above biomarkers of ACC have attracted soaring attention, the relevant research is still in the stage of experimental research or clinical trial. Hence, it is necessary to scout masked bio-targets to forecast the prognosis in the early stage or adopt new treatment strategies.

The centromeric protein F (CENPF), a mitotic centromere protein, is involved in chromosome segregation during mitosis of cell cycle and expressed in a cell cycle-related pattern. The expression peak of CENPF is in the cell cycle of the G2/M phase. The aberrant expression of CENPF has been found in multiple malignancies, including hepatocellular carcinoma (HCC) [19–21], prostate cancer [22], and other carcinomas [23, 24], but little literature about ACC. Specifically, the latest studies have acclaimed that overexpression of CENPF markedly promoted cell proliferation in HCC [20] and induced tumor metastasis in prostate cancer with a poor prognosis [25]. Lin et al. have reported that the signal transduction mediated by CENPF promoted the recurrence and metastasis of prostate cancer [26]. Consequently, CENPF might be a meaningful biomarker for inferring the prognosis of various cancers, including ACC; Moreover, screening the potential molecular mechanisms of CENPF mediated carcinogenic or anti-tumor signaling pathways may furnish new therapeutic strategies for ACC. Glancing at the expression profiles of CENPF in various cancers, we found that CENPF was overexpressed in ACC and negatively correlated with prognosis. However, the expression level, genetic changes, biological process, molecular mechanism, and prognosis of CENPF in ACC have not been further elucidated.

As one of the powerful weapons for biomedical research, the gene-chip and high-throughput sequencing technology has greatly enriched and promoted the research of cancer genomics [27–29]. In this essay, combined with the IHC detection of human samples, cell line experiment, and the analysis of GEO and TCGA databases, it suggested that CENPF was overexpressed, associated with a dismal prognosis, involved in cell cycle, and related signaling pathways in ACC. The results of this study will help to broaden the understanding of ACC, comprehensively analyze the correlation between CENPF and the pathogenesis and progress of ACC, and furnish beneficial clues for the initiation and development of ACC.

## Materials and methods

### Clinical specimens

All Formalin-fixed and paraffin-embedded (PPFE) samples were collected from patients diagnosed as ACC and adrenocortical adenoma and stored at 20–25 °C, from Jan 2011 to Jan 2021 at Taihe Hospital of Hubei University of Medicine, China. According to the pathological characteristics of two pathologists, all ACC patients were diagnosed and graded. Finally, 6 cases of ACC, 12 cases of adrenocortical adenoma (benign tumor), and 12 cases of normal adrenal cortex samples (adjacent to adrenocortical adenoma) were involved in this study. Details of all enrolled patients were listed in Additional file 1: Table S1.

### Histological analysis

Hematoxylin–eosin (HE) and immunohistochemistry (IHC) staining of PPFE samples were conducted following the steps in the manufacturer’s instructions. Specifically, 3 μm of ACC tissues, adrenocortical adenoma tissues, and normal adrenal cortex tissues were sliced from the PPFE. For HE, tissue sections were stained with a hematoxylin–eosin staining kit (E607318-0200, Sangon, Shanghai) for morphological observations. For IHC, all slices were dewaxed with xylene and rehydrated with graded ethanol. The 3% hydrogen peroxide was used to block endogenous peroxidase activity in methanol for 10 min. The sections were incubated with CENPF primary antibody (Additional file 2: Table S2) for 1 h at 37 °C, and incubated with HRP labeled the second antibody at 37 °C for 0.5 h, hematoxylin staining was performed at 37 °C for 30 s. Lastly, CENPF positive cells were tallied in five random high-power fields (400×), and the average positive cell ratio was calculated.

### Bioinformatics data processing

The Gene Expression Omnibus (GEO) of NCBI, an open-access genomics database, was widely used for the systematic analysis of cancer genes. Only two GEO datasets were retrieved for analysis of adrenocortical carcinoma, including GSE90713 and GSE19750. Details of those datasets were listed in Additional file 3: Table S3. The ACC data of TCGA were derived from Genomic Data Commons Data Portal, containing 79 ACC samples.

As an interactive online service platform, Gene Expression Profiling Interactive Analysis (GEPIA) is widely conducted to analyze gene expression of tumors and control samples from the TCGA and GTEx datasets [30]. Thus, the GEPIA dataset was utilized to measure the mRNA expression of CENPF between ACC and normal tissues, and different pathological stages.

### Correlation between CENPF expression and clinicopathological parameters in ACC

The correlation between CENPF and clinicopathological features was studied based on the TCGA-ACC dataset. The79 ACC tissues in the expression matrix were divided into two groups, including 39 ACC tissues with CENPF low expression (CENPF^low^) and 40 ACC samples with CENPF high expression (CENPF^high^) by the median cutoffs. The expression groups were grouped by the 'ggrisk' package of R software (version 4.0.0) [31, 32]. Sanguini diagram was drawn via the ‘ggalluval’ package [33] for displaying the distribution of the gene expression in survival status, ages, genders, stages, and other clinical characteristics for ACC. Then, the univariate (uni-cox) and multivariate cox (multi-cox) regression analyses were analyzed and applied to develop the Nomogram. Then, ‘forestplot’ R packages were applied to present several parameters, including the *p-*values, HR, and 95% confidence interval (CI) by forest [34]. Based on the results of multi-cox analysis, The Nomogram was constructed to provide a graphical representation of the risk factors and calculate the 1-year, 2-year, and 3-year overall recurrence for an ACC patient via 'rms' R package [35].

### Prognosis of CENPF expression in ACC

The prognosis analysis, including overall survival (OS) and progression-free survival (PFS), was performed in ACC with CENPF^high^ and CENPF^low^ by ‘survival’ and ‘survminer’ packages [31]. The timeROC analysis was applied to predict the accuracy of CENPF and risk score by the 'timeROC' package [32], and the AUC threshold is set to 0.80.

### Gene-set enrichment analysis (GSEA)

The GSEA software 4.0.3 (Broad Institute, USA) was applied to probe and uncovered biological mechanisms in TCGA-ACC patients, including 39 CENPF^low^ and 40 CENPF^high^ ACC samples [36, 37]. The four predefined gene-sets, including 'c2.cp.kegg.v7.2.symbols.gmt', 'h.all.v7.2.symbols.gmt', 'c2.cp.go.v7.2.symbols.gmt', and 'c2.cp.biocarta.v7.2.symbols.gmt' were analyzed. Normalized enrichment scores (NES) were reckoned as the main GSEA statistic results. Statistical significance threshold were set as |NES|> 1, normalized p-values (NOM p-values) < 0.05 and FDR < 0.25. Additionally, analysis of protein–protein interaction (PPI) network was performed to explore the interaction between CENPF and related proteins based on The Search Tool for the Retrieval of Interacting Genes (STRING) database (version 11.5).

### Immune cell infiltration signatures

As one of the crucial indicators to speculate the effect of immunotherapy, the immune cell infiltration in tumors has become a research hotspot [38]. CIBERSORTx, the online service platform that estimated the abundance of multiple cell types in the mixed cell population by inputting a standardized gene expression matrix [39], was carried out to analyze immune cell infiltration based on the TCGA-ACC dataset. The results showed the abundance of 22 types of immune cells, including T cells, B cells, natural killer (NK) cells, monocytes, macrophages (Mφ), dendritic cells (DC), and granulocytes (mast cells, eosinophils, and neutrophils). 'Limma' package of R software was performed to normalize the expression matrix from the TCGA dataset. Then, the expression of LAG3, CTLA4, PD-1, PD-L1, and HAVCR2, closely related to immunotherapy, were explored in ACC [40]. To predict the anti-tumor effect of immunosuppressive agents, the 'ggstatsplot' package of R software was further conducted to analyze the correlation between the expression of CENPF and microsatellite instability (MSI) or tumor mutation burden (TMB) [41].

### Gene-drug interaction network

The gene-drug interaction network of CENPF was constructed via Comparative Toxicogenomics Database (CTD) [42] for chemotherapeutic drugs that could reduce or elevate the mRNA or protein expression levels of CENPF, and visualized by the OmicShare tools.

### Human SW13 cells in vitro assay

#### The siRNA experiment

The siRNA transfections were performed according to the manufacturer’s instructions. As one of the human ACC cell lines, SW13 cells were transfected with 50 nmol of CENPF siRNA (siCENPF), CDK1 siRNA (siCDK1), or negative control siRNA (siNC) in a special medium (CM0451, Procell, China) for 48 h. Then, SW13 cells were lysed by TRIzol reagent (Invitrogen, USA) for total RNA isolation. The cDNA was obtained by reverse transcriptase kit (Invitrogen, USA). SYBR Green PCR Mixture (Qiagen, Germany) and specific primers were performed in ABI Prism 7500 analyzer (Applied Biosystems, USA). GAPDH was an endogenous reference gene. Three replicates were set for all reactions. The 2^−ΔΔCt^ method was applied to calculate the relative expression of CENPF or CDK1 in ACC samples. The details of primers were listed in Additional file 4: Table S4.

#### Western blotting

The human SW13 cells were transfected with siRNA targeting CENPF, CDK1, or control siRNA for 48 h. Cell lysates were denatured for SDS-PAGE electrophoresis and then were transferred to PVDF membranes (Millipore, USA). The PVDF membranes were incubated with CENPF or CDK1 antibody (Additional file 2: Table S2) overnight at 4 °C and incubated with the secondary HRP-labeled antibody for 1 h at room temperature. Lastly, the PVDF membranes were visualized by Lumi-Glo reagents (Millipore, USA).

#### Cell proliferation assay

A total of 1 × 10^4^ cells of human SW13 cells were seeded in 96-well plates at 100 μL and transfected with 50 nmol of siCENPF, siCDK1, or siNC, then added 10 μL CCK-8 solution (Beyotime, China) to each well. After incubated for 96 h, the absorbance (optical density, OD), representing the density of cells, was measured at 450 nm.

#### Cell migration assay

Wound healing experiments were performed to analyze cell migration. Human SW13 cells were plated in 12-well plates in DMEM with 10% fetal bovine serum (FBS) and transfected with 50 nmol of siCENPF, siCDK1, or siNC for 48 h. A 20 μL pipette tip was utilized to make wounds. Then each well was washed five times by PBS to remove the floating cells, and 4 mL DMEM (10% FBS, 1% antibiotic–antimycotic) was subjected. The scratch areas were drawn at 0 and 48 h.

#### Cell adhesion assay

Human SW13 cells were seeded in 12-well plates (1 × 10^5^ cells/mL), transfected with 50 nmol of siRNAs for 48 h, and then transferred to 24-well plates for 3 h. All cells were rinsed and fixed with 4% PFA for 20 min. Then, cells were stained by Cristal-violet Staining Kits (Beyotime, China) and incubated for 10 min. Cells adhered to the stroma were photographed and counted by Image J software.

#### Cell invasion assay

Human SW13 cells transfected with siCENPF, CDK1, or siNC for 48 h were plated in TranswellR cell culture chambers (Corning, USA) with 1 × 10^4^ cells/well. The upper chamber of a TranswellR insert was filled with cell suspension. High concentration FBS (20% FBS), as a chemoattractant, was subjected to the lower chamber for 24 h. The cells under the membrane were fixed with 4% PFA and stained with crystal violet. Cells were photographed and counted by Image J software in ten random fields per chamber.

#### Cell apoptosis analyses

Human SW13 cells transfected with siCENPF, CDK1, or siNC for 48 h were disposed with Annexin V-FITC kit (Beyotime Biotechnology, China) and analyzed by flow cytometry (FACSCalibur, Bio-Rad, USA) to detect cell apoptosis. Data were analyzed using FlowJo7.6 software.

#### Cell cycle assay

For cell cycle assay, a cell cycle assay kit (ab112116, Abcam) was purchased. After being transfected with siCENPF, CDK1, or siNC for 48 h, SW13 cells were harvested and fixed in 70% ice-cold ethanol overnight at 4 °C. Then cells were analyzed by the Cell Cycle Assay Kit (ab112116, Abcam) in a flow cytometer. Lastly, data were analyzed by FlowJo7.6 software.

### Statistical analysis

Statistical analysis was conducted via SPSS 22.0 (IBM SPSS Inc. Chicago, IL) or GraphPad Prism 9.0 (San Diego, CA) software. The results are expressed as mean ± standard deviation (SD). Comparison of two groups was performed by two-tail Student’s *t-*test or one-way analysis of variance. The Kruskal–Wallis test was used to assess the correlation between gene expression and clinical characteristics. The univariate (uni-cox) and multivariate cox (multi-cox) regression models were used to analyze the effect of CENPF expression on survival time and other clinical characteristics (stage, grade, etc.). Kaplan–Meier survival curves were compared using the log-rank test. The Pearson’s correlation test was performed to examine the correlation between two parameters. p < 0.05 was considered as a significant difference (ns = no significance, *p < 0.05,**p < 0.01,***p < 0.001).

## Results

### CENPF is overexpressed in ACC, but not in adrenocortical adenoma or normal adrenal cortex tissues

**Fig. 1:**
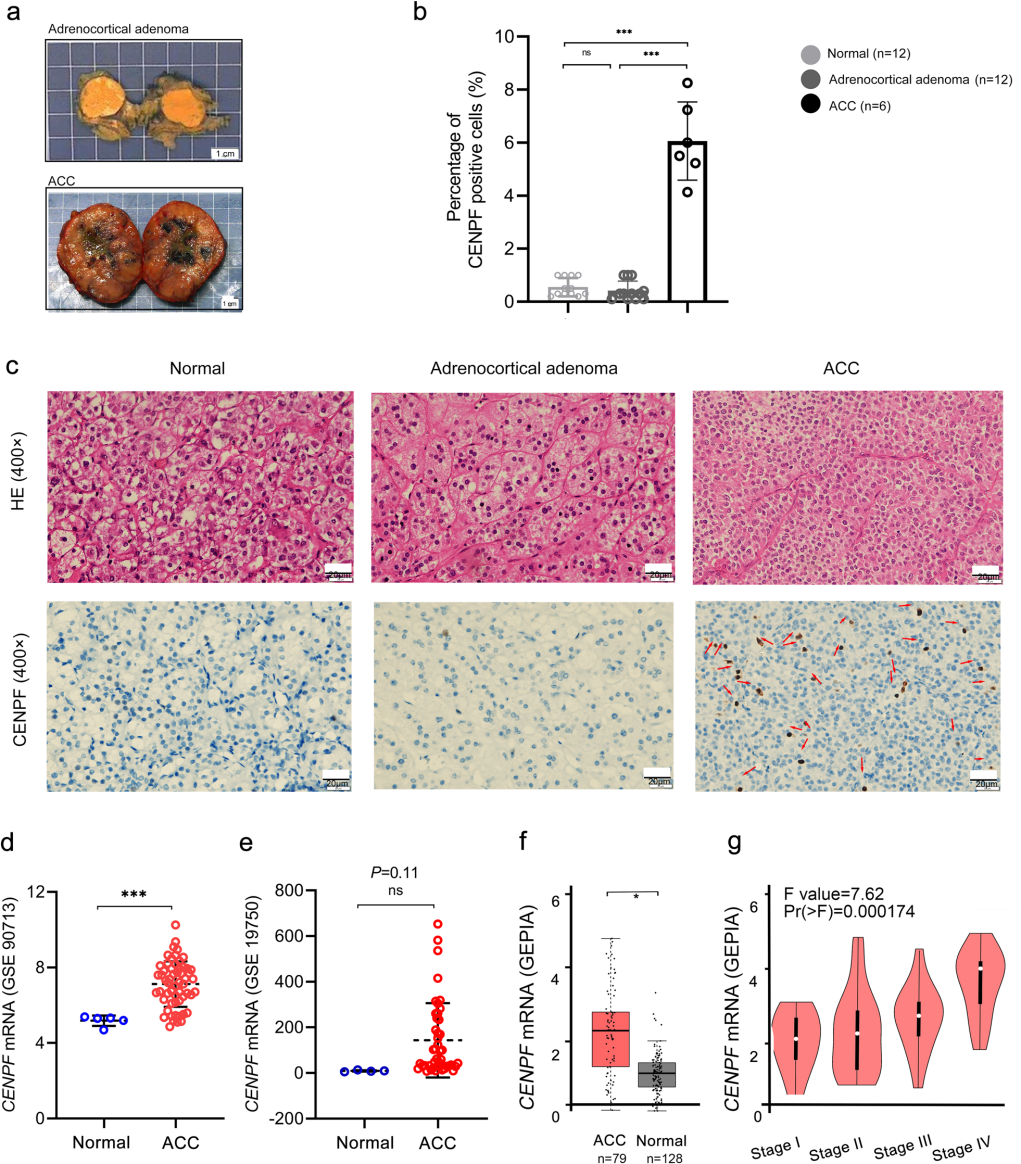
CENPF was overexpressed in ACC patients, compared with normal adrenocortical samples or adrenocortical adenoma. **a** Gross specimen photography for adrenocortical adenoma (upper) and ACC (bottom). **b** Percentage of CENPF positive cells in ACC via IHC (high power fields, 400×). **c** IHC for HE (upper) and CENPF protein expression (bottom) in the normal adrenal cortex, adrenocortical adenoma, and ACC samples (high power fields, 400×). The expression of CENPF was denoted by the red arrows. The expression of CENPF from **d** GSE90713 and **e** GSE19750. **f** Relative expression of CENPF in ACC, normal tissue, and in tumor stage I–IV (**g**) based on GEPIA online server

Significant differences were found in morphology and histology among normal adrenal cortex, adrenocortical adenoma (benign tumor), and ACC (malignant tumor). Firstly, gross specimen photography showed that for adrenocortical adenoma, the nodule section was golden and hard; For ACC, the capsule was intact or defective, some areas were gray red, and necrotic (Fig. 1a). Results of IHC-P showed that CENPF was mostly upregulated in ACC compared with adrenocortical adenoma samples or normal adrenal cortex tissues, but no significant difference was discovered between adrenocortical adenoma and normal tissues (p < 0.001, Fig. 1b, c). Furthermore, the results were validated in public databases, including GEO and TCGA datasets, for ACC patients. The mRNA expression of CENPF was dramatically overexpressed in GSE90713 (p < 0.01, Fig. 1d). But interestingly, there was no significant difference in the expression of this gene in GSE19750 (p = 0.11, Fig. 1e). This might be in case of too small sample size in normal adrenocortical tissues (n = 4). Meanwhile, the controversy attracted us to further explore. Moreover, correlation analysis by the GEPIA database showed that the mRNA expression of CENPF was evidently overexpressed in ACC tumors compared with that in normal ones (p < 0.001, Fig. 1f), and was positively correlated with the tumor stage for ACC (p < 0.001, Fig. 1g).

**Fig. 2:**
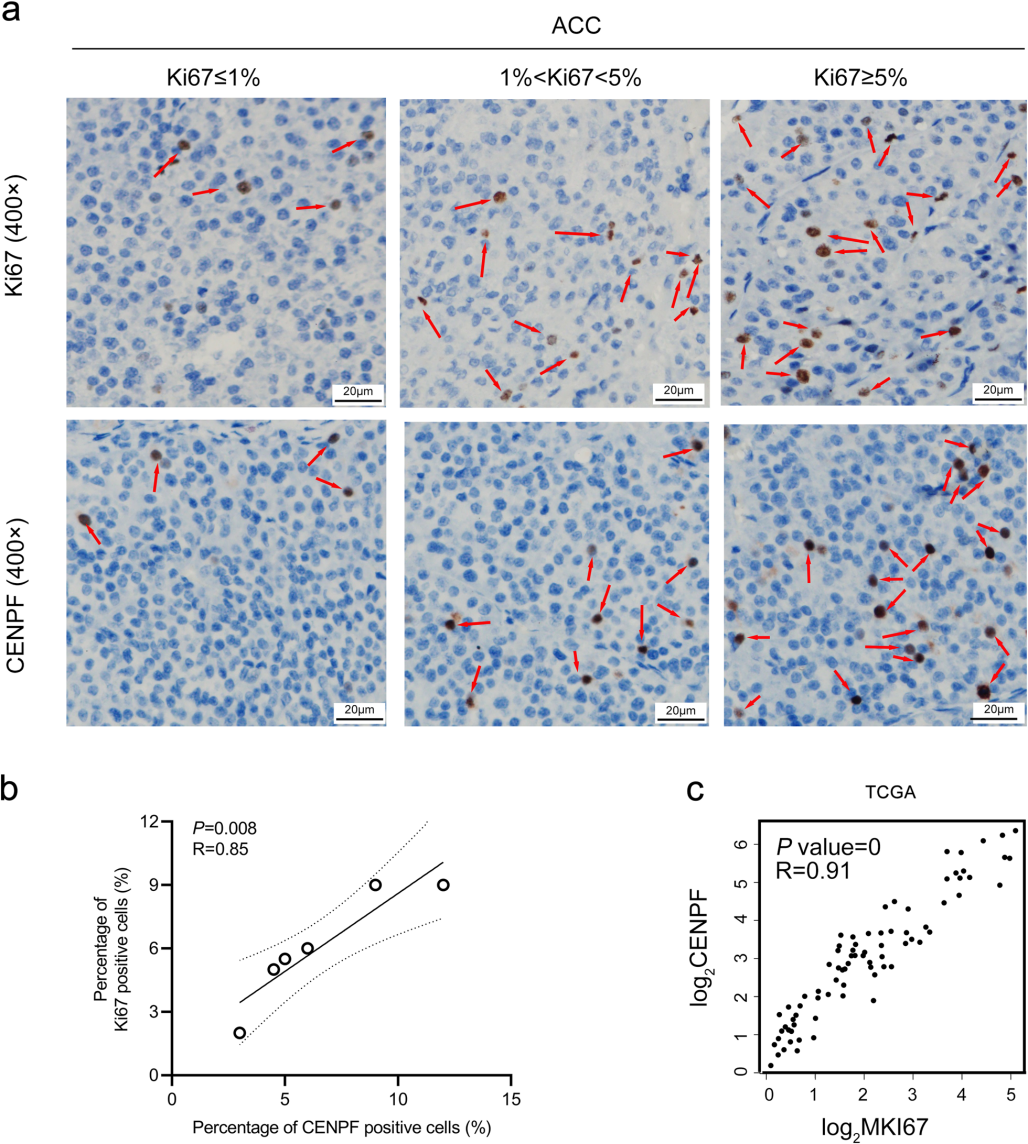
The expression of CENPF was positively related to the cell proliferation index, Ki67. **a**, **b** The proportion of CENPF positive cells was positively correlated with the proportion of Ki67 positive cells (n = 6). **c** Based on TCGA-ACC, the expression of CENPF was positively correlated with that of MKI67 at the RNA level. The expression of CENPF or Ki67 was depicted by the red arrows

Additionally, Ki67, as a cell proliferation index, was investigated in ACC. According to the proportion of Ki67 positive cells in ACC, the relationship between the expression of *CENPF* and cell proliferation was further discussed. As result, the expression of CENPF, namely the proportion of CENPF positive cells, was positively correlated with the proportion of Ki67 positive cells (p = 0.008, R = 0.85, Fig. 2a, b). Moreover, the expression of CENPF was positively correlated with that of MKI67 expression based on the TCGA-ACC dataset (p = 0, R = 0.91, Fig. 2c). It indicated that the expression of CENPF was closely related to the cell proliferation in patients with ACC.

### Analysis of CENPF in clinicopathological parameters for ACC patients

**Fig. 3:**
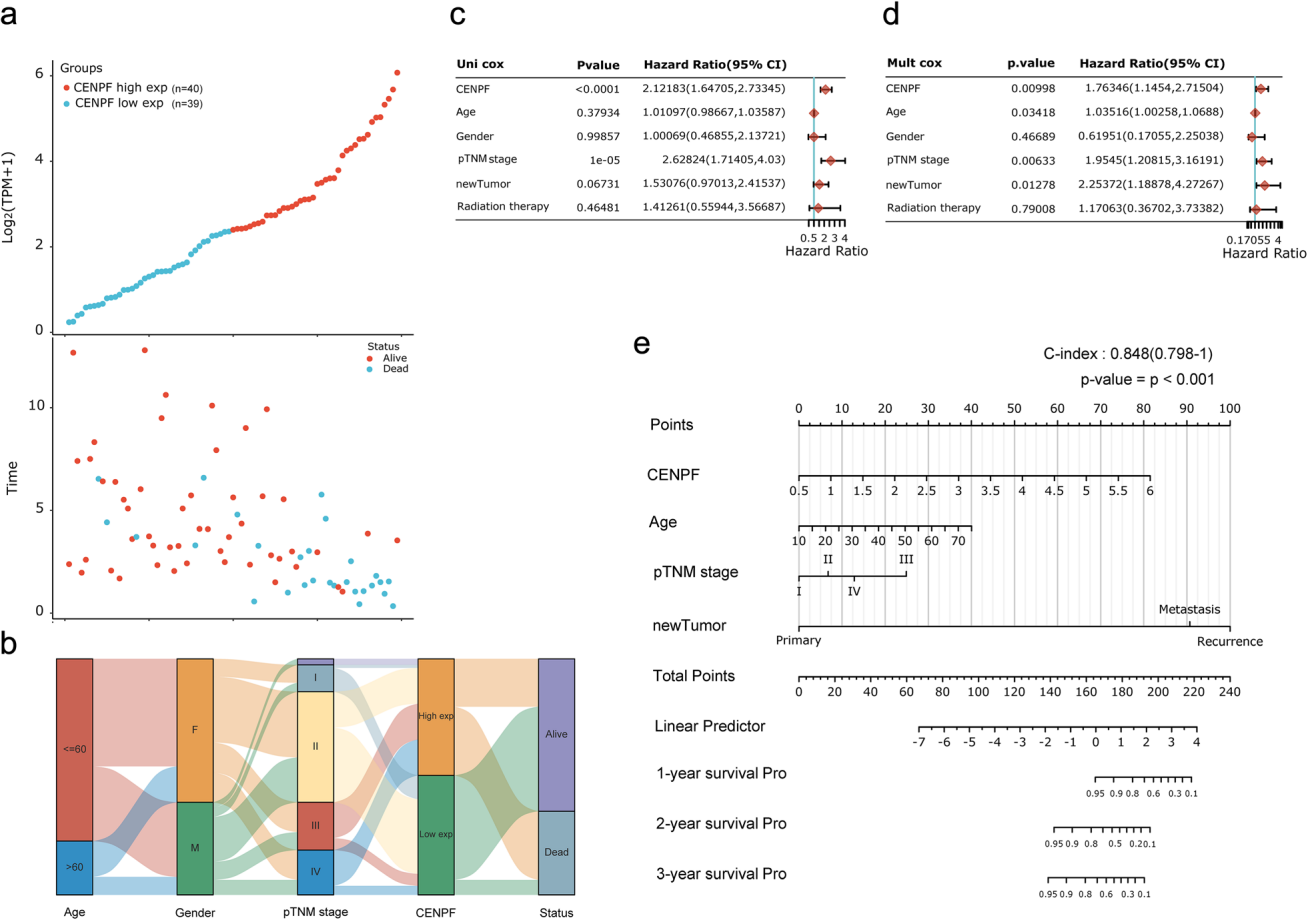
Prognosis analysis of CENPF in clinicopathological parameters for ACC patients. **a** CENPF expression (high or low) and survival status (alive or dead) of ACC patients. All the sample order is consistent. **b** Sanguini diagram for depicting the distribution of CENPF expression in age, gender, pTNM stage, and survival status. The P-value and hazard ratio of CENPF and associated parameters of the uni-cox (**c**) and multi-cox (**d**) analysis. **e** Nomogram for assessing the survival rate of 1-year, 2- year, and 3-year in ACC patients related to CENPF expression

For figuring out the correlation between CENPF expression and clinicopathological parameters in ACC, all ACC patients were divided into two subgroups, including 39 CENPF^low^ and 40 CENPF^high^ samples according to the median cutoffs (Fig. 3a). As presented in Table 1, the expression of CENPF was not correlated with age, gender, laterality, and mitotane therapy (p > 0.05). Compared with CENPF^low^, ACC patients with CENPF^high^ suffered manifestly advanced pathological stages (especially stage III and IV, p < 0.001). In tumor status, new events, or residual tumor, more ACC cases with CENPF^high^ were prone to lose the chance of surgery, relapse, or form residual tumor than CENPF^low^ (p < 0.001). Additionally, the CENPF^high^ group showed a higher Weiss score (especially Weiss score 6–9, p = 0.002). Moreover, the sanguini diagram described the distribution of CENPF in age, gender, pTNM stage, and status (Fig. 3b). Further, the correlation between CENPF and relevant clinical parameters, including age, gender, pTNM stage, etc. on the OS of ACC patients were identified via the uni-cox and multi-cox regression analysis. As result, the CENPF expression and pTNM stage were closely related to the OS of ACC patients in uni-cox analysis (Fig. 3c, all p < 0.05), and the *CENPF* expression and pTNM stage could be served as independent prognostic factors for ACC patients in multi-cox analysis (Fig. 3d, all p < 0.05). Finally, the survival rate of 1-year, 2- year, or 3- year in one ACC patient related to high CENPF expression was evaluated by Nomogram (Fig. 3e).

**Table 1:** Correlation between CENPF expression and clinicopathological characteristics

Characteristic	CENPF expression level		p-value
	Low (n = 39)	High (n = 40)	
Age, n (%)			0.571
≤ 50	22 (27.8%)	19 (24.1%)	
> 50	17 (21.5%)	21 (26.6%)	
Gender, n (%)			0.196
Female	27 (34.2%)	21 (26.6%)	
Male	12 (15.2%)	19 (24.1%)	
Pathologic stage, n (%)			< 0.001
Stage I	8 (10.4%)	1 (1.3%)	
Stage II	25 (32.5%)	12 (15.6%)	
Stage III	4 (5.2%)	12 (15.6%)	
Stage IV	2 (2.6%)	13 (16.9%)	
Tumor status, n (%)			< 0.001
Tumor free	28 (36.4%)	11 (14.3%)	
With tumor	11 (14.3%)	27 (35.1%)	
New events, n (%)			< 0.001
No	28 (36.8%)	11 (14.5%)	
Yes	11 (14.5%)	26 (34.2%)	
Residual tumor^a^, n (%)			< 0.001
R0	38 (54.3%)	17 (24.3%)	
R1	0 (0%)	6 (8.6%)	
R2	0 (0%)	9 (12.9%)	
Laterality, n (%)			0.436
Left	20 (25.3%)	25 (31.6%)	
Right	19 (24.1%)	15 (19%)	
Mitotane therapy, n (%)			0.259
No	16 (21.3%)	10 (13.3%)	
Yes	22 (29.3%)	27 (36%)	
Weiss score, n (%)			0.002
2	3 (4.9%)	0 (0%)	
3	9 (14.8%)	2 (3.3%)	
4	7 (11.5%)	2 (3.3%)	
5	5 (8.2%)	2 (3.3%)	
6	3 (4.9%)	5 (8.2%)	
7	5 (8.2%)	4 (6.6%)	
8	1 (1.6%)	8 (13.1%)	
9	0 (0%)	5 (8.2%)	

^a^Residual tumor: R0, no residual tumor; R1, residual tumor under the microscope; R2, residual tumor under-eye field

### Overexpressed CENPF is associated with dismal survival in ACC patients

**Fig. 4:**
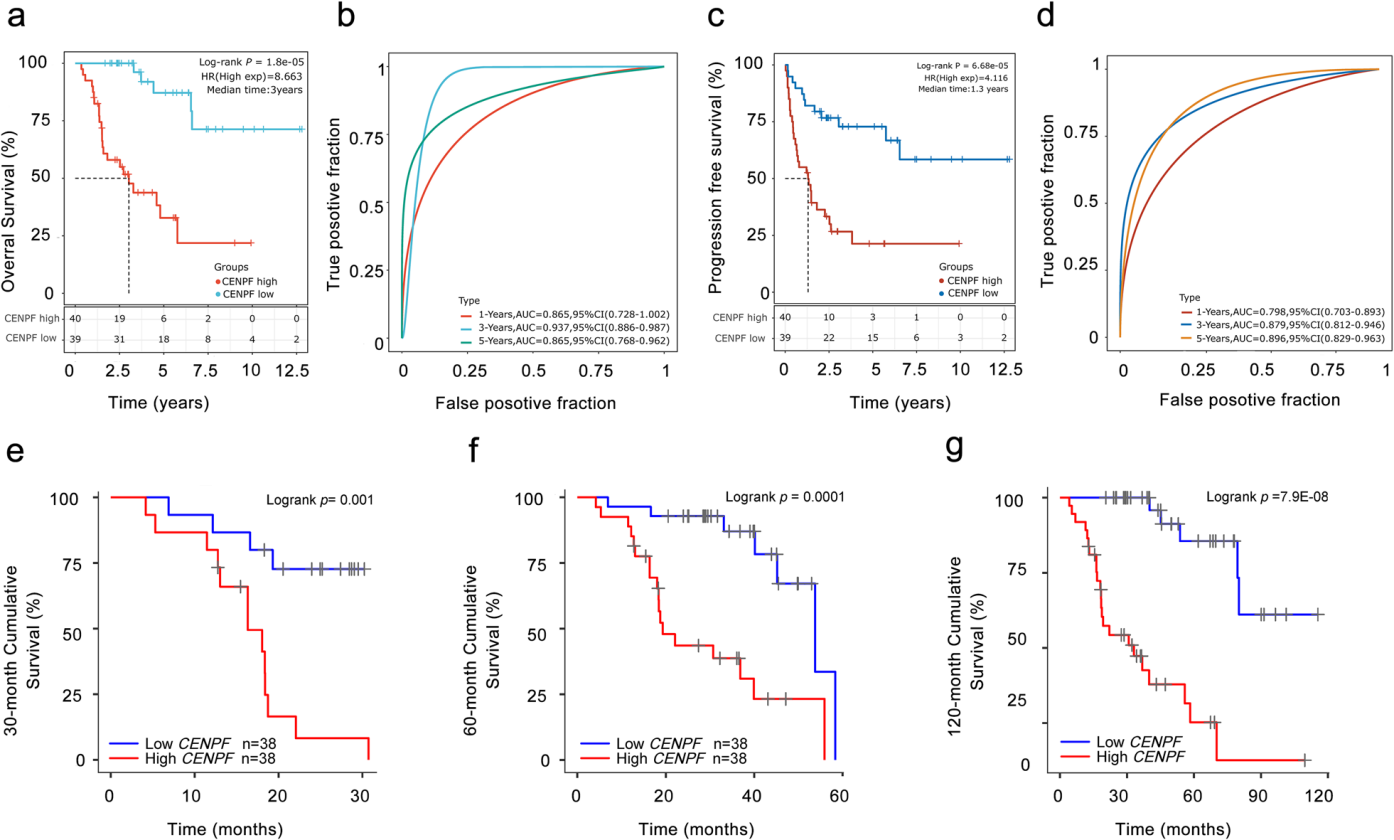
The prognosis of CENPF expression in ACC patients. The OS (**a**), ROC curves of CENPF at 1-year, 3-year, and 5-year for OS (**b**), PFS (**c**), ROC curves of CENPF at 1-year, 3-year, and 5-year for PFS (**d**), the survival rate of 30-month (**e**), 60-month (**f**) and 120-month (**g**) for patients with CENPF^high^ and CENPF^low^ in ACC at the threshold value of p < 0.05. In time-dependent ROC analysis, the higher the AUC value, the stronger the predictive ability of the gene

According to the survival rate analysis, upregulation of CENPF expression predicts poor OS (HR = 8.66, log-rank p = 1.8e−05; Fig. 4a) and PFS (HR = 4.11, log-rank p = 6.68e−05; Fig. 4c) in ACC. ROC curve analyses demonstrated that the AUC of CENPF at 1-year, 3-year, and 5-year for OS (Fig. 3b) and PFS (Fig. 3d) pointed out that the expression of CENPF has a good predictive effect on the prognosis of ACC (all AUC value > 0.75). Additionally, subgroup analyses indicated that overexpression of CENPF in ACC patients acted as a risk factor for 30-month, 60-month, and 120-month OS (all log-rank p ≤ 0.001, Fig. 4e–g).

### GSEA analyses reveal masked molecular mechanisms of CENPF in tumorigenesis and progression

**Fig. 5:**
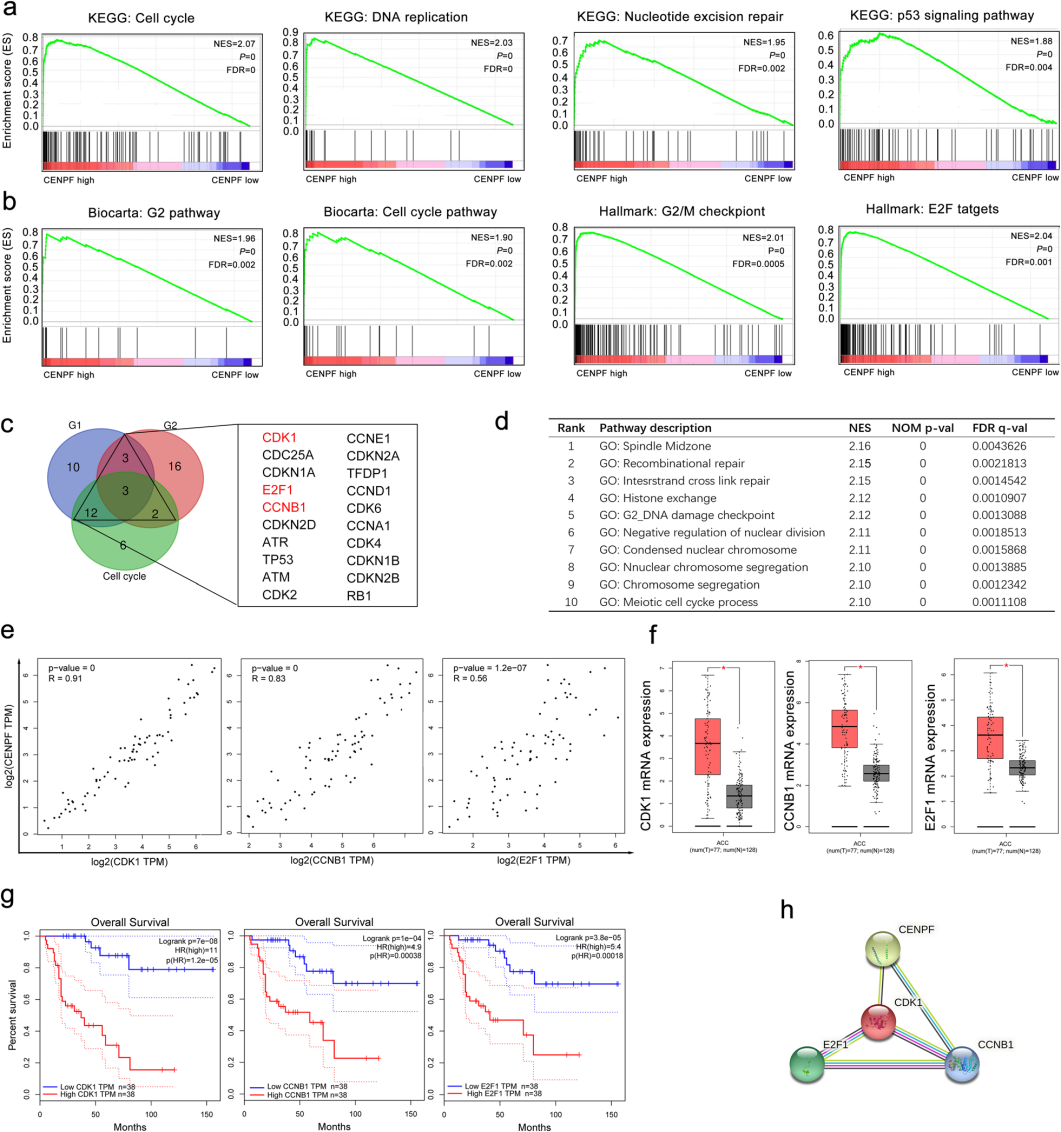
CENPF regulates the cell cycle by interacting with CDK1, E2F1, and CCNB1 in ACC. **a** GSEA analysis of the KEGG pathway showed that cell cycle, DNA replication, nucleotide excision repair, and p53 signaling pathway were overtly involved in ACC with CENPF^high^. **b** GSEA analysis of Biocarta pathway and Hallmark description suggested that overexpressed CENPF was positively related to cell cycle (especially G2-phase of mitosis), G2/M checkpoint, and E2F targets. **c** Venn’s graph demonstrated that 20 genes, including CDK1, E2F1, and CCNB1, were overlapped in at least two groups. **d** Top 10 GSEA analysis of GO terms. **e** Correlation analysis between CDK1, CCNB1, E2F1, and CENPF expression in ACC. **f** Expression of CDK1 and E2F1 in ACC and normal samples. **g** Survival analysis of CDK1, CCNB1, and E2F1 in ACC based on GEPIA database. **h** PPI network figured out the close interactions among CENPF, CDK1, and CCNB1

To uncover the potential roles of CENPF in cancer-related signaling pathways, GSEA was carried out to explain the gene expression profiles of ACC samples with CENPF^low^ and CENPF^high^. According to GSEA analysis of the KEGG pathway, CENPF^high^ patients were mainly enriched in cell cycle, DNA replication, nucleotide excision repair, and p53 signaling pathway, etc. (Fig. 5a, Additional file 5: Table S5). Then, GSEA enrichment analyses of Biocarta pathway and Hallmark description suggested that cell cycle (NES = 2.03, p = 0; especially G2-phase of mitosis, NES = 1.96, *p* = 0), G2/M checkpoint (NES = 2.01, p = 0), and E2F targets (NES = 2.04, p = 0) were mostly enriched in ACC patients with CENPF^high^, which implied that CENPF might regulate cell cycle by interacting with E2Fs proteins (Fig. 5b, Additional file 6: Table S6, Additional file 7: Table S7, and Additional file 8: Table S8). Additionally, relevant studies have proclaimed that E2F1 played the role as a transcription factor of CENPF in the NCI-60 cell line [43] and regulated the cell cycle of the G2/M-phase transition [44, 45]. Thus, we supposed that CENPF may regulate the cell cycle by interacting with E2F1 in ACC. Venn graph suggested that 20 genes (CDK1, CDC25A, CDKN1A, E2F1, CCNB1, CDKN2D, ATR, TP53, ATM, CDK2, CCNE1, CDKN2A, TFDP1, CCND1, CDK6, CCNA1, CDK4, CDKN1B, CDKN2B and RB1) were co-expressed in at least two groups (Fig. 5c). It has been reported that CENPF mainly regulated the spindle separation in mitosis [46]. As listed in Fig. 5d and Additional file 7: Table S7, “spindle midzone” was the top 1 term in GESA analysis of GO terms. By screening the correlation, expression, and prognosis of these 20 genes, we picked out that CDK1, CCNB1and E2F1 were mostly overexpressed in ACC samples compared with normal ones (Fig. 5f, all p < 0.05), and positively relevant with the expression of CENPF (Fig. 5e, all p < 0.01). The overexpressed CDK1, CCNB1, and E2F1 also were related to the negative OS of ACC patients (Fig. 5g, all p < 0.01). In addition, the PPI network revealed that close interactions were found among CENPF, CDK1, and CCNB1 (Fig. 5h). Thus, it suggested that CENPF regulated the cell cycle by interacting with CDK1, E2F1, and CCNB1 in ACC. The overexpressed CENPF might play pivotal roles in the regulation of the G2/M-phase transition mediated cell cycle in the progression of ACC.

### CENPF interference affects the regulation of cell cycle in SW13 cells

**Fig. 6:**
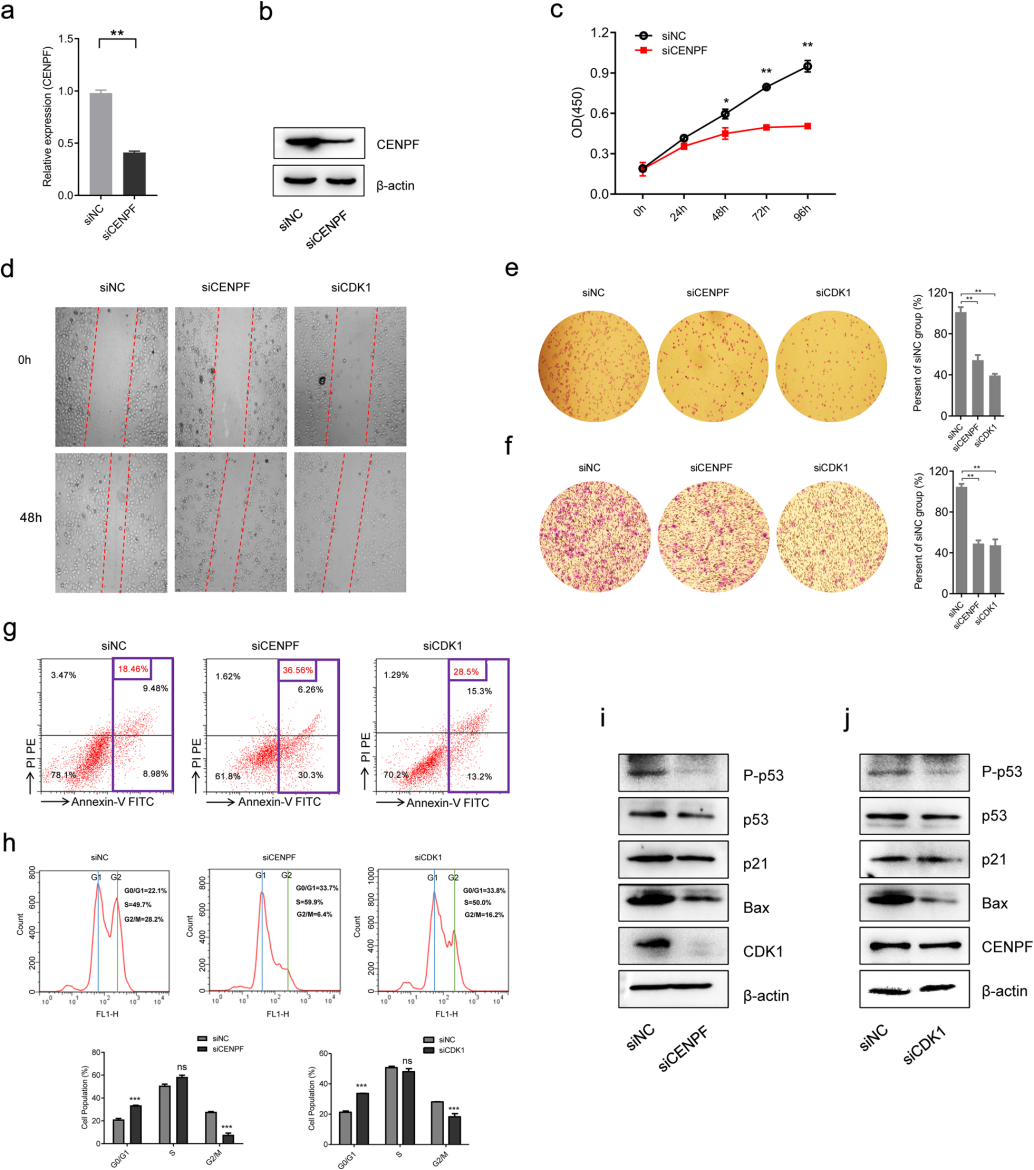
Coupled with CDK1, CENPF is involved in cell proliferation, cell migration, cell cycle, and cell apoptosis of SW13 cells. Detection method by **a** The qRT-PPCR and **b** Western blotting for CENPF expression in human SW13 cells after transfected with siCENPF, or siNC for 48 h. **c** Cell proliferation assay for human SW13 cells after cells were transfected with siCENPF, or siNC for 96 h. **d** Wound healing experiment for cell mobility investigation; The red line denotes the migration ability of SW13 cells transfected with siRNAs for 48 h. **e** Cell adhesion assay of SW13 cells transfected with siRNAs for 48 h. **f** Cell invasion assay of SW13 cells transfected with siRNAs for 48 h. **g** Apoptosis and **h** cell cycle analysis of SW13 cells transfected with siRNAs for 48 h. **i** Western blotting for CDK1, p53, P-p53, p21, and Bax in human SW13 cells transfected with siCENPF or siNC for 48 h. **j** Western blotting for CENPF, p53, P-p53, p21, and Bax in human SW13 cells transfected with siCDK1 or siNC for 48 h

To further search the role of CENPF in ACC*,* CENPF siRNAs (siCENPF) were conducted via ACC cell line, human SW13 cells, as in vitro experiments. The expression of CENPF was overtly inhibited in siCENPF, compared with siNC Fig. 6a, b). In cell proliferation assay, CENPF interference evidently inhibited cell proliferation (Fig. 6c) and cell migration (Fig. 6d) in human SW13 cells after cells were transfected for 48 h. Inhibition of CENPF distinctly reduced adhesion between tumor cells and matrix (Fig. 6e) and cell invasion (Fig. 6f) in human SW13 cells. In addition, flow cytometry assay proclaimed that down-regulation of CENPF overtly intensified cell apoptosis, including early and late stages of cell apoptosis (18.46% vs 36.56%, Fig. 6g). Then, CENPF interference induced cell cycle arrest at the G2/M-phase transition in human SW13 cells (28.2% vs 6.4%, Fig. 6h). As analyzed above in Fig. 5, the expression of CENPF was closely related to CDK1 in ACC. Therefore, SiCDK1 was designed to probe the correlation between CENPF and CDK1 (Additional file 9: Fig. S1a, b). Down-regulation of CDK1 significantly suppressed cell proliferation (Additional file 9: Fig. S1c) and cell mobility (Fig. 6d) in human SW13 cells after cells were transfected 48 h. Down-regulation of CDK1 significantly restrained adhesion between tumor cells and matrix (Fig. 6e) and cell invasion (Fig. 6f) in human SW13 cells. Additionally, the flow cytometry experiment depicted that down-regulation of CDK1 overtly augmented cell apoptosis, including early and late stages of cell apoptosis (18.46% vs 28.5%, Fig. 6g). Lastly, CDK1 interference blocked the cell cycle at the G2/M-phase transition in human SW13 cells (28.2% vs 16.2%, Fig. 6h).

### CENPF regulates CDK1 expression and p53 signaling in human SW13 cells

The molecular mechanism of CENPF was further explored in SW13 cells. As represented in Fig. 6, inhibition of the expression of CENPF resulted in the decrease of CDK1 expression Fig. 6i), while inhibition of CDK1 had no significant effect on the expression of CENPF (Fig. 6j). It suggested that CENPF might regulate the expression of CDK1. Furthermore, the GSEA enrichment analysis disclosed that the p53 signaling pathway might serve as a masked molecular mechanism of CENPF involved in the occurrence and development of human ACC (Fig. 5a). Therefore, the phosphorylation of p53 (P-p53), the expression levels of p53, p21, and Bax were detected by Western blotting in SW13 cells. As the interference of CENPF and CDK1 by siRNA, the expression levels of P-p53, p21, and Bax were significantly decreased compared with the siNC in SW13 cells (Fig. 6i, j). Therefore, inhibition of CENPF downregulated the expression of CDK1, suppressed the phosphorylation of p53, and altered downstream protein levels, including apoptosis and cell cycle-related proteins.

### The potential therapeutic strategies for the therapy of ACC

**Fig. 7:**
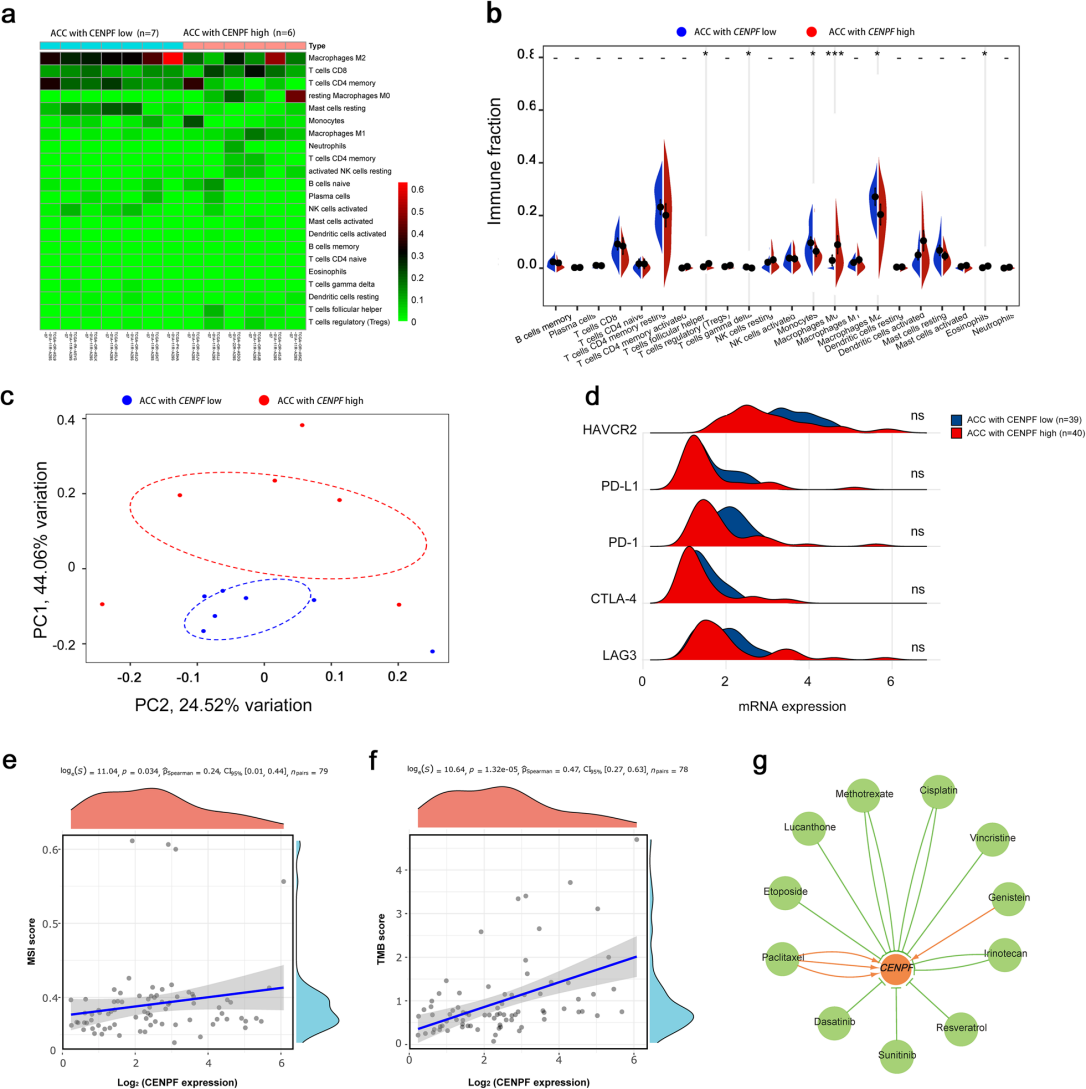
The potential therapeutic strategies or available chemical drugs for the treatment of ACC. **a** Immune infiltration analysis of CENPF^high^ (n = 6) and CENPF^low^ (n = 7) samples in ACC. The immune fraction (**b**) and PCA analysis (**c**) in ACC samples. **d** Screening the expression level between the five genes, including LAG3, CTLA4, HAVCR2, PD-1 and PD-L1, and CENPF in ACC. Correlation between CENPF expression and MSI (**e**) and TMB (**f**) in ACC. The horizontal axis represents the gene expression distribution, and the vertical axis denotes the expression distribution of the TMB/MSI score. The density curve on the right side represents the distribution trend of the TMB/MSI score; The density curve of the upper part represents the distribution trend of genes; The value represents the correlation p-value, correlation coefficient, and calculation method. **g** The gene-drug interaction network of CENPF and related chemotherapeutics. Red arrows: chemotherapeutics up-regulate CENPF expression; green arrows: chemotherapeutics down-regulate CENPF expression. The numbers of arrows between the chemotherapeutics and CENPF in this network denote the supported numbers of previous literature

Next, the available chemical drugs and treatment strategies, including immunotherapy, that may be favorable for the treatment of ACC were further explored. Firstly, the immune cell infiltration in ACC with CENPF^high^ and CENPF^low^ were analyzed to investigate immunotherapy. All the 79 ACC tissues from the TCGA database were divided into two groups, including 40 CENPF^high^ and 39 CENPF^low^ tissues by the median value. CIBERSORTx was applied to analyze immune cell infiltration, including 22 cell types with low and high expression of CENPF. p < 0.05 was statistically significant. The normalized by 'Limma' packages and then filtered groups including 7 CENPF^low^ and 6 CENPF^high^ ACC tissues were identified for further analysis. The expression of 22 immune cell populations for each sample was presented as heatmaps (Fig. 7a). The immune enrichment score was dramatically various between 6 CENPF^high^ and 7 CENPF^low^ samples. As shown in Fig. 7b, follicular helper T cells, M0 macrophages, Eosinophils were mainly enriched in ACC with CENPF^high^, compared with the CENPF^low^ group. However, the immune enrichment score of gamma delta T cells, monocytes, and M2 macrophages were much lower in CENPF^high^, compared with the CENPF^low^ group. The principal component analysis (PCA) showed that manifest difference was probed in immune infiltration between CENPF^high^ and CENPF^low^ ACC samples (Fig. 7c). It might indicate that the immune heterogeneity of the CENPF was significant in ACC patients. Otherwise, the mRNA expression of LAG3, CTLA4, PD-1, PD-L1, and HAVCR2, tightly related to tumor immunosuppression or tolerance, were not significant differences in ACC with CENPF^high^, compared with CENPF^low^ (Fig. 7d, all p > 0.05). Moreover, the correlation between the expression of CENPF and MSI or TMB was analyzed to unraveled that CENPF expression was significantly associated with MSI score (Fig. 7e, p = 0.034) and TMB score (Fig. 7f, p < 0.01) in ACC. It showed that overexpression of CENPF might augment the accumulation of abnormal gene mutation, namely TMB, in the process of DNA replication in ACC. Then, the gene-drug interaction network indicated that a variety of drugs could affect the expression levels of CENPF in mRNA or protein levels (Fig. 7g). For example, Cisplatin, Sunitinib, and Etoposide could restrain CENPF expression level while Paclitaxel and Genistein could induce CENPF expression level. Generally, all these CENPF inhibitors were deemed as potential targets for the treatment of ACC.

## Discussion

Accumulative kinds of literature have asserted that CENPF is closely related to cell proliferation as well as tumorigenesis [19, 20, 46]. In recent years, shreds of evidence have arisen that the overexpression of CENPF was a frequent behavior in different malignant tumors and was closely related to the tumor deterioration and dismal prognosis in multiple neoplasms, including HCC [19, 20], breast cancer [23], prostate cancer [22] and other tumors [24]. In prostate cancer*,* CENPF has been proved to be a primary regulatory factor of prostate cancer and a bleak prognostic predictor of survival and metastasis [25]. Nevertheless, the expression alteration, molecular mechanisms, and biological functions of CENPF in ACC are still unclear. This essay aimed to systematically study the expression patterns, prognosis, and latent functions of CENPF in ACC.

In human ACC samples, the IHC staining showed that CENPF was significantly overexpressed and positively correlated with cell proliferation index, Ki67. It suggested that hyper-expression of CENPF may serve a pivotal role in the process of cell proliferation. Compared with single array analysis, comprehensive analysis of multiple arrays has been considered as a rational and feasible method to analyze the reliability of results [47]. Thus, further insight into the expression of CENPF was gained via assaying ACC-related datasets from GEO and TCGA. It confirmed that *CENPF* was upregulated in ACC, compared with normal ones, and positively correlated with pathological stage. The uni-cox and multi-cox analysis suggested that the CENPF expression and pTNM stage were correlated with the OS of ACC patients and might serve as independent prognostic factors in ACC patients. Thus, it suggested that CENPF could be viewed as a biomarker to make a distinction between ACC and adrenocortical adenoma or normal adrenal cortex tissues. Then, the prognosis of CENPF was evaluated to announce that overexpression of CENPF was overtly related to the poor OS and PFS in ACC patients. Overexpression of CENPF was associated with poor 30-month, 60-month, and 120-month OS. Our results were reinforced by the number of publications. Huang Y et al*.* asserted that up-regulation of CENPF was a hazard factor for the prognosis of HCC [21]. A recent study suggested that overexpressed CENPF exerted as a cancer-driver gene in the formation and development of human cancers [48]. Consequently, our results showed that the expression of *CENPF* was upregulated in ACC and might play a crucial role in the tumorigenesis of ACC. Then, the GSEA enrichment analysis of the KEGG pathway indicated that CENPF^high^ was mainly involved in the cell cycle, p53 signaling pathway, and DNA replication. Moreover, the GSEA analyses of the Biocarta pathway and Hallmark description uncovered that CENPF regulated the G2/M-phase cell cycle by interacting with CDK1, E2F1*,* and CCNB1 in human ACC.

Based on the above results, the siRNA system was established to further explore potential biological functions and molecular mechanisms by performing in vitro experiments in ACC cell line, human SW13 cells. It showed that interference of CENPF inhibited the cell proliferation, adhesion between tumor cells and matrix, cell migration, cell invasion, and the G2/M-phase transition, while remarkably enhancing cell apoptosis of SW13 cells. These results indicated that CENPF might induce the G2/M-phase transition by interacting with CDK1, which played a vital role in promoting cell proliferation. And, the overexpression of CENPF induced cell migration, cell invasion, and inhibiting cell apoptosis. By western blotting, interference of CENPF and CDK1 by siRNA, the phosphorylation of p53 (P-p53), the expression levels of p21, and Bax were decreased compared with the siNC in SW13 cells. Therefore, the CENPF upregulated the expression level of CDK1, the phosphorylation of p53, and altered downstream protein levels, including apoptosis and cell cycle-related proteins.

**Fig. 8:**
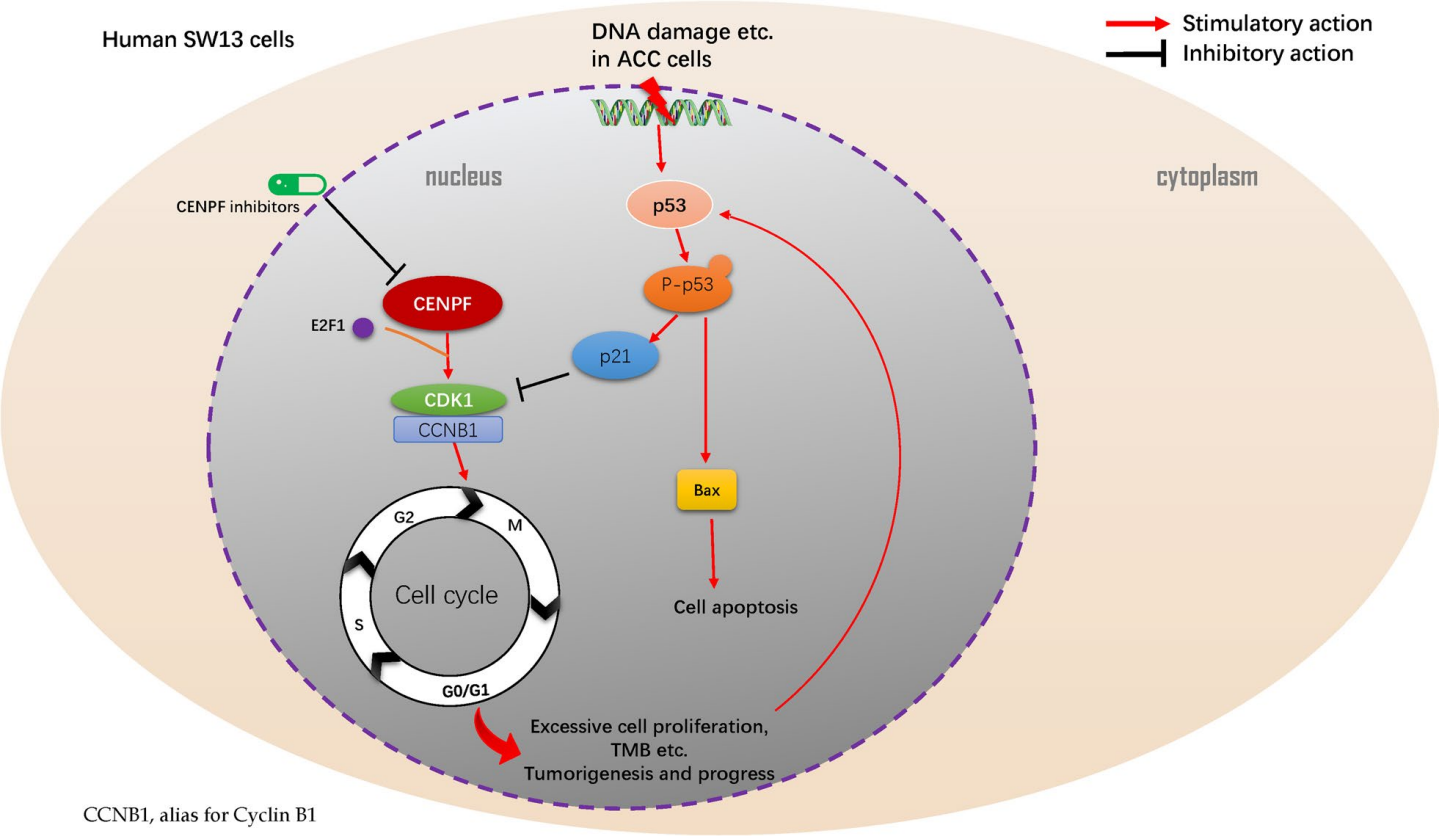
Mechanism diagram. Overexpressed CENPF could induce the expression of CDK1, augment G2/M-phase conversion, promote cell proliferation and lead to the accumulation of abnormal gene mutations, namely TMB, during the process of DNA replication or cell cycle. Meanwhile, these tumor signatures, including DNA damage, etc., might trigger the activation of the p53 signaling pathway and the downstream expression of p21 and Bax, thus inducing p53 mediated anti-tumor effect by arresting the cell cycle of G2/M-phase and boosting cell apoptosis in ACC

Previous literature reports enhanced our results. Kojima K et al. demonstrated that CDK1 inhibitor enhanced p53 mediated mitochondrial apoptosis by Bax activation and the G2/M-phase cell cycle arrest in acute myeloid leukemia [49]. Chen S et al. corroborated that the activation of p53 and p21 can inhibit the expression of CDKs, E2Fs, and other factors that promote DNA replication in the cell cycle of G1/S-phase arrest in bladder cancer [50]. Danupon N et al. reported that CCNB1/CDK1 complex was relocated to mitochondria during the G2/M phase to phosphorylate and activate p53 at Ser-315, thus inducing an anti-apoptotic response in HCT116 cells [51]. Bowen et al. asserted that CCNB1/CDK1 regulated mitochondrial energy metabolism, promoted cell cycle progression and tumor response to radiotherapy [52, 53]. Abnormal mitosis induced by CCNB1/CDK1 complex is an enormous element of cancer development or progression [54]. Combined with previous related research, we draw the conclusion that overexpression of CENPF upregulated CDK1 mediated G2/M-phase transition, cell proliferation, cell migration, and invasion. Meanwhile, overexpression of CENPF activated p53 mediated anti-tumor effect by inducing P-p53, p21 mediated G2/M-phase cell cycle arrest, or Bax-mediated cell apoptosis in ACC (Fig. 8).

Although a variety of biomarkers or underlying molecular mechanisms have been reported, there is still a lack of effective drugs for ACC patients [55]. Accordingly, it is necessary to further investigate the potential target genes or immunotherapy related to the high expression of CENPF. Firstly, the immune microenvironment has been viewed as one of the hallmarks of multiple cancer [56]. In this study, the immune cell population of ACC with CENPF^high^ and CENPF^low^ was analyzed by CIBERSORTx to show that the cell subtypes were significantly different in ACC samples of CENPF^high^, compared with CENPF^low^. Specifically, follicular helper T cells, M0 macrophages, Eosinophils were enriched in ACC with CENPF^high^, compared with CENPF^low^. Moreover, patients with high CENPF expression characterized higher TMB score and MSI score. Additionally, the expression of LAG3, CTLA4, HAVCR2, PD-1 and PD-L1*,* closely related to immunosuppression, were not evidently different in ACC patients of CENPF^high^ group and CENPF^low^ group. The result indicated that overexpression of CENPF might accumulate TMB and MSI in the process of DNA replication. Moreover, gene-drug interaction network was constructed by CTD and found that multiple CENPF inhibitors, including Cisplatin, Sunitinib, and Etoposide, might act as beneficial targets for the treatment of ACC. However, identification and verification of these assumption are still demanded further molecular mechanisms, pharmaceutical or clinical trials in the future.

In this paper, we have discussed that overexpressed CENPF was closely related to the occurrence and development of ACC, indicating that CENPF might be acted as promising prognostic markers or therapeutic targets for ACC. However, many limitations still existed in our research. Firstly, as a rare disease with few cases, the ACC sample size of clinical specimens and the public databases are relatively small. Then, the clinical correlation, prognosis, and potential molecular mechanism of CENPF in ACC patients are mainly analyzed based on the TCGA database; Thus, this study lacks some of our own clinical information, such as tumor subtypes, follow-up data and, expression profiles of CENPF-related genes, which may bring limitations to the clinical application value of CENPF in ACC. Lastly, the in vitro siRNA cell line experiment has a certain exploration of the biological function of CENPF, but in vivo experiments or further research for ACC patients are still essential. Thus, it is still necessary to further perform in vivo experiments or accumulate sufficient clinical samples to verify the above conclusions.

## Conclusion

In summary, this study pointed out that CENPF is significantly overexpressed in ACC patients. The overexpressed CENPF predicted a poor prognosis of ACC and might augment the progress of ACC. The results showed that CENPF might consider as a prognostic biomarker or therapeutic target for ACC patients. According to in vitro experiments, overexpression of CENPF upregulated CDK1 mediated the G2/M-phase transition, cell proliferation, cell migration, cell invasion, and accumulation of TMB and MSI. Moreover, overexpression of CENPF might activate the p53 signaling pathway, which exerted potential anti-tumor effects. In brief, the study highlighted the accumulating evidence about CENPF and related signaling pathways, which might provide beneficial enlightenment for the development of CENPF-mediated therapeutic drugs or formulation of strategies for the individualized treatment of ACC patients.

## Supplementary Information

**Additional file 1: Table S1.** Details of all patients involved in this study.

**Additional file 2: Table S2.** Details of all antibodies involved in this study.

**Additional file 3: Table S3.** Details of ACC studies and associated microarray datasets from GEO database.

**Additional file 4: Table S4.** The sequences information of qRT-PCR primers and siRNAs.

**Additional file 5: Table S5.** GSEA analysis of KEGG pathway.

**Additional file 6: Table S6.** GSEA enrichment analyses of Biocarta pathway.

**Additional file 7: Table S7.** GESA analysis of GO term.

**Additional file 8: Table S8.** GSEA enrichment analyses of Hallmark description.

**Additional file 9: Fig. S1.** CDK1 interference in human SW13 cells. (a) The qRT-PCR and (b) Western blotting for CDK1 expression in human SW13 cells after cells were transfected with siCDK1, or siNC for 48 h. (c) Cell proliferation assay for human SW13 cells after cells were transfected with siCDK1 or siNC for 96 h.

## Data Availability

All publicly available datasets analyzed in this study can be acquired from GEO (https://www.ncbi.nlm.nih.gov/geo/) and TCGA (https://portal.gdc.cancer.gov/).

## Abbreviations

ACC: Adrenocortical carcinoma
CENPF: Centromere protein F
TCGA: The Cancer Genome Atlas
OS: Overall survival
PFS: Progression-free survival
GEO: Gene expression omnibus
ROC: Receiver operator characteristic
qRT-PCR: Reverse transcription-quantitative PCR
IHC: Immunohistochemistry
CCK-8: Cell counting kit-8
GSEA: Gene set enrichment analysis
GO: Gene Ontology
KEGG: Kyoto Encyclopedia of Genes and Genomes
CCND1: Cyclin D1
FDR: False discovery rate
SD: Standard deviation

## References

[1] Crona J, Beuschlein F (2019). Adrenocortical carcinoma—towards genomics guided clinical care. Nat Rev Endocrinol.

[2] Henley SJ, Ward EM, Scott S, Ma J, Anderson RN, Firth AU (2020). Annual report to the nation on the status of cancer, part I: national cancer statistics. Cancer.

[3] Kebebew E, Reiff E, Duh QY, Clark OH, McMillan A (2006). Extent of disease at presentation and outcome for adrenocortical carcinoma: have we made progress?. World J Surg.

[4] Fassnacht M, Johanssen S, Quinkler M, Bucsky P, Willenberg HS, Beuschlein F (2009). Limited prognostic value of the 2004 International Union Against Cancer staging classification for adrenocortical carcinoma: proposal for a revised TNM classification. Cancer.

[5] Fojo T, Huff L, Litman T, Im K, Edgerly M, Del Rivero J (2020). Metastatic and recurrent adrenocortical cancer is not defined by its genomic landscape. BMC Med Genomics.

[6] Herrmann LJ, Heinze B, Fassnacht M, Willenberg HS, Quinkler M, Reisch N (2012). TP53 germline mutations in adult patients with adrenocortical carcinoma. J Clin Endocrinol Metab.

[7] Pinto EM, Chen X, Easton J, Finkelstein D, Liu Z, Pounds S (2015). Genomic landscape of paediatric adrenocortical tumours. Nat Commun.

[8] Zheng S, Cherniack AD, Dewal N, Moffitt RA, Danilova L, Murray BA (2016). Comprehensive pan-genomic characterization of adrenocortical carcinoma. Cancer Cell.

[9] Sbiera S, Leich E, Liebisch G, Sbiera I, Schirbel A, Wiemer L (2015). Mitotane inhibits sterol-O-acyl transferase 1 triggering lipid-mediated endoplasmic reticulum stress and apoptosis in adrenocortical carcinoma cells. Endocrinology.

[10] Tissier F, Cavard C, Groussin L, Perlemoine K, Fumey G, Hagneré AM (2005). Mutations of beta-catenin in adrenocortical tumors: activation of the Wnt signaling pathway is a frequent event in both benign and malignant adrenocortical tumors. Cancer Res.

[11] Lippert J, Appenzeller S, Liang R, Sbiera S, Kircher S, Altieri B (2018). Targeted molecular analysis in adrenocortical carcinomas: a strategy toward improved personalized prognostication. J Clin Endocrinol Metab.

[12] Le DT, Uram JN, Wang H, Bartlett BR, Kemberling H, Eyring AD (2015). PD-1 blockade in tumors with mismatch-repair deficiency. N Engl J Med.

[13] Mohan DR, Lerario AM, Else T, Mukherjee B, Almeida MQ, Vinco M (2019). Targeted assessment of G0S2 methylation identifies a rapidly recurrent, routinely fatal molecular subtype of adrenocortical carcinoma. Clin Cancer Res.

[14] Koschmann C, Calinescu AA, Nunez FJ, Mackay A, Fazal-Salom J, Thomas D (2016). ATRX loss promotes tumor growth and impairs nonhomologous end joining DNA repair in glioma. Sci Transl Med.

[15] Brondani VB, Lacombe AMF, Mariani BMP, Montenegro L, Soares IC, Bezerra-Neto JE (2021). Low protein expression of both ATRX and ZNRF3 as novel negative prognostic markers of adult adrenocortical carcinoma. Int J Mol Sci.

[16] Juhlin CC, Goh G, Healy JM, Fonseca AL, Scholl UI, Stenman A (2015). Whole-exome sequencing characterizes the landscape of somatic mutations and copy number alterations in adrenocortical carcinoma. J Clin Endocrinol Metab.

[17] Svahn F, Paulsson JO, Stenman A, Fotouhi O, Mu N, Murtha TD (2018). TERT promoter hypermethylation is associated with poor prognosis in adrenocortical carcinoma. Int J Mol Med.

[18] Taylor DR, Ghataore L, Couchman L, Vincent RP, Whitelaw B, Lewis D (2017). A 13-steroid serum panel based on LC-MS/MS: use in detection of adrenocortical carcinoma. Clin Chem.

[19] Dai Y, Liu L, Zeng T, Zhu YH, Li J, Chen L (2013). Characterization of the oncogenic function of centromere protein F in hepatocellular carcinoma. Biochem Biophys Res Commun.

[20] Yang X, Miao BS, Wei CY, Dong RZ, Gao PT, Zhang XY (2019). Lymphoid-specific helicase promotes the growth and invasion of hepatocellular carcinoma by transcriptional regulation of centromere protein F expression. Cancer Sci.

[21] Huang Y, Chen X, Wang L, Wang T, Tang X, Su X (2021). Centromere protein F ( CENPF ) serves as a potential prognostic biomarker and target for human hepatocellular carcinoma. J Cancer.

[22] Shahid M, Lee MY, Piplani H, Andres AM, Zhou B, Yeon A (2018). Centromere protein F (CENPF), a microtubule binding protein, modulates cancer metabolism by regulating pyruvate kinase M2 phosphorylation signaling. Cell Cycle.

[23] Sun J, Huang J, Lan J, Zhou K, Gao Y, Song Z (2019). Overexpression of CENPF correlates with poor prognosis and tumor bone metastasis in breast cancer. Cancer Cell Int.

[24] Varis A, Salmela AL, Kallio MJ (2006). Cenp-F (mitosin) is more than a mitotic marker. Chromosoma.

[25] Aytes A, Mitrofanova A, Lefebvre C, Alvarez MJ, Castillo-Martin M, Zheng T (2014). Cross-species regulatory network analysis identifies a synergistic interaction between FOXM1 and CENPF that drives prostate cancer malignancy. Cancer Cell.

[26] Lin SC, Kao CY, Lee HJ, Creighton CJ, Ittmann MM, Tsai SJ (2016). Dysregulation of miRNAs-COUP-TFII-FOXM1-CENPF axis contributes to the metastasis of prostate cancer. Nat Commun.

[27] Sealfon SC, Chu TT (2011). RNA and DNA microarrays. Methods Mol Biol.

[28] Ritchie ME, Phipson B, Wu D, Hu Y, Law CW, Shi W (2015). limma powers differential expression analyses for RNA-sequencing and microarray studies. Nucleic Acids Res.

[29] Shi J, Chen L, Chen Y, Lu Y, Chen X, Yang Z (2019). Aldo-keto reductase family 1 member B10 (AKR1B10) overexpression in tumors predicts worse overall survival in hepatocellular carcinoma. J Cancer.

[30] Tang Z, Li C, Kang B, Gao G, Li C, Zhang Z (2017). GEPIA: a web server for cancer and normal gene expression profiling and interactive analyses. Nucleic Acids Res.

[31] Zhang Z, Lin E, Zhuang H, Xie L, Feng X, Liu J (2020). Construction of a novel gene-based model for prognosis prediction of clear cell renal cell carcinoma. Cancer Cell Int.

[32] Lin W, Wu S, Chen X, Ye Y, Weng Y, Pan Y (2020). Characterization of hypoxia signature to evaluate the tumor immune microenvironment and predict prognosis in glioma groups. Front Oncol.

[33] Zeng D, Li M, Zhou R, Zhang J, Sun H, Shi M (2019). Tumor microenvironment characterization in gastric cancer identifies prognostic and immunotherapeutically relevant gene signatures. Cancer Immunol Res.

[34] Xiong Y, Yuan L, Xiong J, Xu H, Luo Y, Wang G (2020). An outcome model for human bladder cancer: a comprehensive study based on weighted gene co-expression network analysis. J Cell Mol Med.

[35] Jeong SH, Kim RB, Park SY, Park J, Jung EJ, Ju YT (2020). Nomogram for predicting gastric cancer recurrence using biomarker gene expression. Eur J Surg Oncol.

[36] Subramanian A, Tamayo P, Mootha VK, Mukherjee S, Ebert BL, Gillette MA (2005). Gene set enrichment analysis: a knowledge-based approach for interpreting genome-wide expression profiles. Proc Natl Acad Sci.

[37] Mootha VK, Lindgren CM, Eriksson K-F, Subramanian A, Sihag S, Lehar J (2003). PGC-1α-responsive genes involved in oxidative phosphorylation are coordinately downregulated in human diabetes. Nat Genet.

[38] Galon J, Costes A, Sanchez-Cabo F, Kirilovsky A, Mlecnik B, Lagorce-Pagès C (2006). Type, density, and location of immune cells within human colorectal tumors predict clinical outcome. Science.

[39] Newman AM, Steen CB, Liu CL, Gentles AJ, Chaudhuri AA, Scherer F (2019). Determining cell type abundance and expression from bulk tissues with digital cytometry. Nat Biotechnol.

[40] Wu F, Li GZ, Liu HJ, Zhao Z, Chai RC, Liu YQ (2020). Molecular subtyping reveals immune alterations in IDH wild-type lower-grade diffuse glioma. J Pathol.

[41] Thorsson V, Gibbs DL, Brown SD, Wolf D, Bortone DS, Ou Yang TH (2018). The immune landscape of cancer. Immunity.

[42] Davis AP, Grondin CJ, Johnson RJ, Sciaky D, McMorran R, Wiegers J (2019). The comparative toxicogenomics database: update 2019. Nucleic Acids Res.

[43] Reinhold WC, Erliandri I, Liu H, Zoppoli G, Pommier Y, Larionov V (2011). Identification of a predominant co-regulation among kinetochore genes, prospective regulatory elements, and association with genomic instability. PLoS ONE.

[44] Clijsters L, Hoencamp C, Calis JJA, Marzio A, Handgraaf SM, Cuitino MC (2019). Cyclin F controls cell-cycle transcriptional outputs by directing the degradation of the three activator E2Fs. Mol Cell.

[45] Chen Q, Wang L, Jiang M, Huang J, Jiang Z, Feng H (2018). E2F1 interactive with BRCA1 pathway induces HCC two different small molecule metabolism or cell cycle regulation via mitochondrion or CD4+T to cytosol. J Cell Physiol.

[46] Hussein D, Taylor SS (2002). Farnesylation of Cenp-F is required for G2/M progression and degradation after mitosis. J Cell Sci.

[47] Ma T, Liang F, Oesterreich S, Tseng GC (2017). A joint bayesian model for integrating microarray and RNA sequencing transcriptomic data. J Comput Biol.

[48] Kim HE, Kim DG, Lee KJ, Son JG, Song MY, Park YM (2012). Frequent amplification of CENPF, GMNN and CDK13 genes in hepatocellular carcinomas. PLoS ONE.

[49] Kojima K, Shimanuki M, Shikami M, Andreeff M, Nakakuma H (2009). Cyclin-dependent kinase 1 inhibitor RO-3306 enhances p53-mediated Bax activation and mitochondrial apoptosis in AML. Cancer Sci.

[50] Chen S, Zhou Q, Guo Z, Wang Y, Wang L, Liu X (2020). Inhibition of MELK produces potential anti-tumour effects in bladder cancer by inducing G1/S cell cycle arrest via the ATM/CHK2/p53 pathway. J Cell Mol Med.

[51] Nantajit D, Fan M, Duru N, Wen Y, Reed JC, Li JJ (2010). Cyclin B1/Cdk1 phosphorylation of mitochondrial p53 induces anti-apoptotic response. PLoS ONE.

[52] Chen LT, Martinelli E, Cheng AL, Pentheroudakis G, Qin S, Bhattacharyya GS (2020). Pan-Asian adapted ESMO Clinical Practice Guidelines for the management of patients with intermediate and advanced/relapsed hepatocellular carcinoma: a TOS-ESMO initiative endorsed by CSCO, ISMPO, JSMO, KSMO MOS and SSO. Ann Oncol.

[53] Xie B, Wang S, Jiang N, Li JJ (2019). Cyclin B1/CDK1-regulated mitochondrial bioenergetics in cell cycle progression and tumor resistance. Cancer Lett.

[54] Fang L, Du WW, Awan FM, Dong J, Yang BB (2019). The circular RNA circ-Ccnb1 dissociates Ccnb1/Cdk1 complex suppressing cell invasion and tumorigenesis. Cancer Lett.

[55] Dimri M, Satyanarayana A (2020). Molecular signaling pathways and therapeutic targets in hepatocellular carcinoma. Cancers.

[56] Hanahan D, Weinberg RA (2011). Hallmarks of cancer: the next generation. Cell.

