# Supplementary material for: CENPF/CDK1 signaling pathway enhances the progression of adrenocortical carcinoma by regulating the G2/M-phase cell cycle
Source: J Transl Med. 2022 Feb 5;20:78. doi: 10.1186/s12967-022-03277-y (PMC8818156; doi:10.1186/s12967-022-03277-y)
Supplement: Supplementary file 2 — Additional file 2: Table S2. Details of all antibodies involved in this study. [file 12967_2022_3277_MOESM2_ESM.docx]

**Additional file 2: Table S2. Details of all antibodies involved in this study.**

| **Antibodies for western blotting** | **Source** | **Catalogs NO.** | **Molecular weight** |
| --- | --- | --- | --- |
| β-actin Antibody | Santa Cruz | Cat:sc-47778 | 42 kDa |
| Anti-CENPF antibody (for western blotting) | Affinity | Cat:DF2310 | 330 kDa |
| Anti-CDK1 antibody | ZEN BIO | Cat:383884 | 34 kDa |
| Anti-P-p53 antibody (Ser-392) | Bimake | Cat: A5112 | 53 kDa |
| Anti-p53 antibody | Proteintech | Cat:60283-2-Ig | 53 kDa |
| Anti-Bax antibody | Proteintech | Cat:60267-1-Ig | 21 kDa |
| Anti- p21 antibody | Proteintech | Cat:10355-1-AP | 21 kDa |
| Anti-CENPF antibody (for IHC/IF) | Abcam | Cat: ab223847 | 330 kDa |
