# Supplementary material for: CENPF/CDK1 signaling pathway enhances the progression of adrenocortical carcinoma by regulating the G2/M-phase cell cycle
Source: J Transl Med. 2022 Feb 5;20:78. doi: 10.1186/s12967-022-03277-y (PMC8818156; doi:10.1186/s12967-022-03277-y)
Supplement: Supplementary file 3 — Additional file 3: Table S3. Details of ACC studies and associated microarray datasets from GEO database. [file 12967_2022_3277_MOESM3_ESM.docx]

**Additional file 3: Table S3. Details of ACC studies and associated microarray datasets from GEO database.**

| **GEO Series** | **Contributor(s)** | **Sample** | | **Platform** | **Submission time** | **Country** |
| --- | --- | --- | --- | --- | --- | --- |
|  |  | **Tumor** | **Normal** |  |  |  |
| GSE90713 | Farber JM et al, 2016 | 58 | 5 | GPL15207 Affymetrix Human Gene Expression Array | 2016 | USA |
| GSE19750 | Bussey KJ et al, 2010 | 44 | 4 | GPL570 Affymetrix Human Genome U133 Plus 2.0 Array | 2010 | USA |
