# Supplementary material for: CENPF/CDK1 signaling pathway enhances the progression of adrenocortical carcinoma by regulating the G2/M-phase cell cycle
Source: J Transl Med. 2022 Feb 5;20:78. doi: 10.1186/s12967-022-03277-y (PMC8818156; doi:10.1186/s12967-022-03277-y)
Supplement: Supplementary file 4 — Additional file 4: Table S4. The sequences information of qRT-PCR primers and siRNAs. [file 12967_2022_3277_MOESM4_ESM.docx]

**Additional file 4: Table S4.** The sequences information of qRT-PCR primers and siRNAs.

| **Gene name** | **Primers (5**'-**3**') | |
| --- | --- | --- |
| **CENPF** | Forward: AGCACTGATCACCTGTTAGC | Reverse: ACCCACATACAAACAGAGATTG |
| **CDK1** | Forward: TTACAAAGATCAAGGGCTGTCCGCAACA | Reverse: AAAGCCAAGATAAGCAACTCCTTCAGT |
| **GAPDH** | Forward: CGGAGTCAACGGATTTGGTCGTAT | Reverse: AGCCTTCTCCATGGTGGTGAAGAC |
| **siCENPF** | Forward: GACCCAGAAACUAGCUUAUTT | Reverse: AUAAGCUAGUUUCUGGGUCTT |
| **SiCDK1** | Forward: GTACTGCAATTCGGGAAAT | Reverse: AGGCAGACGUGACACGUUATT |
| **siNC** | Forward: UUCUCCGAACGUGUCACGUTT | Reverse: ACGUGACACGUUCGGAGAATT |

siCENPF: CENPF siRNA, siNC: negative control siRNA.
