# Supplementary material for: CENPF/CDK1 signaling pathway enhances the progression of adrenocortical carcinoma by regulating the G2/M-phase cell cycle
Source: J Transl Med. 2022 Feb 5;20:78. doi: 10.1186/s12967-022-03277-y (PMC8818156; doi:10.1186/s12967-022-03277-y)
Supplement: Supplementary file 9 — Additional file 9: Fig. S1. CDK1 interference in human SW13 cells. (a) The qRT-PCR and (b) Western blotting for CDK1 expression in human SW13 cells after cells were transfected with siCDK1, or siNC for 48 h. (c) Cell proliferation assay for human SW13 cells after cells were transfected with siCDK1 or siNC for 96 h. [file 12967_2022_3277_MOESM9_ESM.docx]

**Fig. S1**

**
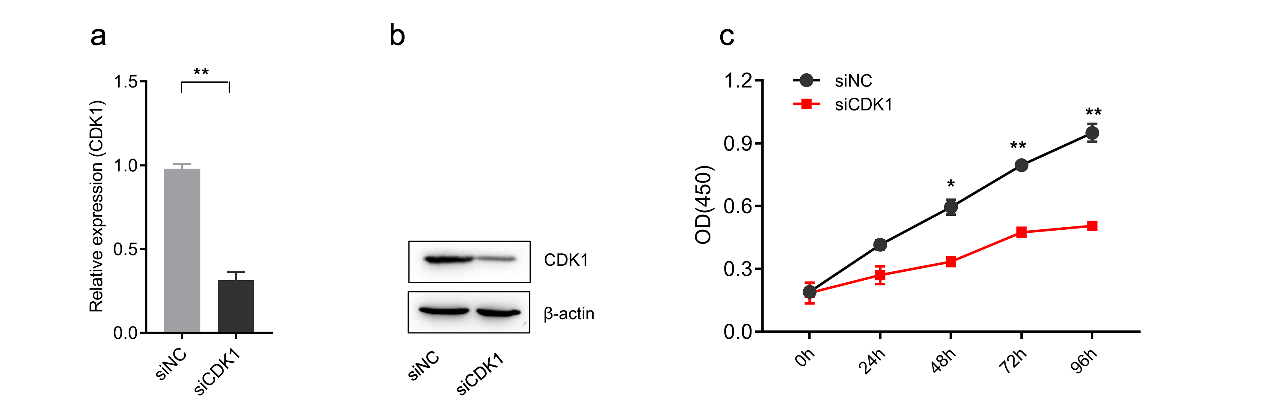
**

**Additional file 9: Fig. S1.** CDK1 interference in human SW13 cells. (a) The qRT-PCR and (b) Western blotting for CDK1 expression in human SW13 cells after cells were transfected with siCDK1, or siNC for 48 hours. (c) Cell proliferation assay for human SW13 cells after cells were transfected with siCDK1 or siNC for 96 hours.
